# Supplementary material for: Clinical and Molecular Differences Suggest Different Responses to Immune Checkpoint Inhibitors in Microsatellite-Stable Solid Tumors with High Tumor Mutational Burden
Source: Cancers (Basel). 2025 Aug 16;17(16):2673. doi: 10.3390/cancers17162673 (PMC12384169; doi:10.3390/cancers17162673)
Supplement: Supplementary file 1 [file cancers-17-02673-s001.zip › Supplementary Table S1 Pathways.pdf]

**Characteristic**	**[SD,<6m]/PD** N = 51[1]	**CR/PR/[SD,>6m]** N = 54[1]	**p-value**[2]	**q-value**[3]
TP53 pathway	45 (88%)	44 (81%)	0.3	>0.9
RTK/RAS Pathway	50 (98%)	52 (96%)	>0.9	>0.9
Cell Cycle pathway	33 (65%)	35 (65%)	>0.9	>0.9
PI3K pathway	35 (69%)	41 (76%)	0.4	>0.9
NOTCH pathway	33 (65%)	38 (70%)	0.5	>0.9
WNT pathway	20 (39%)	22 (41%)	0.9	>0.9
NRF2 pathway	14 (27%)	9 (17%)	0.2	>0.9
TGFβ pathway	11 (22%)	13 (24%)	0.8	>0.9
MYC pathway	14 (27%)	6 (11%)	0.033	0.3
HIPPO pathway	13 (25%)	14 (26%)	>0.9	>0.9

[1]n (%)

[2]Pearson's Chi-squared test; Fisher's exact test

[3]False discovery rate correction for multiple testing
