# Supplementary material for: Clinical and Molecular Differences Suggest Different Responses to Immune Checkpoint Inhibitors in Microsatellite-Stable Solid Tumors with High Tumor Mutational Burden
Source: Cancers (Basel). 2025 Aug 16;17(16):2673. doi: 10.3390/cancers17162673 (PMC12384169; doi:10.3390/cancers17162673)
Supplement: Supplementary file 1 [file cancers-17-02673-s001.zip › Supplementary Table S2 Genes.pdf]

| Characteristic | **[SD,<6m]/PD** N = 51[1] | **CR/PR/[SD,>6m]** N = 54[1] | **p-value**[2] | **q-value**[3] |
|---|---|---|---|---|
| tert | 15 (29%) | 27 (50%) | 0.031 | >0.9 |
| mll2 | 24 (47%) | 13 (24%) | 0.014 | >0.9 |
| fgf19 | 7 (14%) | 1 (1.9%) | 0.028 | >0.9 |
| pold1 | 6 (12%) | 0 (0%) | 0.011 | >0.9 |
| fgf3 | 5 (9.8%) | 0 (0%) | 0.024 | >0.9 |
| abl1 | 7 (14%) | 0 (0%) | 0.005 | >0.9 |
| mtor | 2 (3.9%) | 9 (17%) | 0.033 | >0.9 |

[1]n (%)

[2]Pearson's Chi-squared test; Fisher's exact test

[3]False discovery rate correction for multiple testing
